# Resection of the primary tumor improves the prognosis of gastrointestinal neuroendocrine neoplasms with liver metastases: mutual validation based on SEER database and institutional data

**DOI:** 10.1186/s12876-023-03041-6

**Published:** 2023-11-23

**Authors:** Yifan Liu, Zhixiong Wang, Qi Lin, Ruizhe Cui, Wei Tang, Guanghua Li, Zhao Wang

**Affiliations:** Department of Gastrointestinal Surgery, First Affiliated Hospital of Sun Yat-sen University, Zhongshan 2nd Street, No. 58, 510080, Guangzhou, 86 Guangdong China

**Keywords:** Neuroendocrine neoplasm, SEER, Gastrointestinal, Liver metastasis, Primary tumor resection

## Abstract

**Background:**

Gastrointestinal Neuroendocrine Neoplasms (GI-NENs) often result in liver metastases, and the role of Primary Tumor Resection (PTR) in managing GI-NENs with liver metastases (GI-NENLM) is still debated. This study aimed to investigate the potential benefits of PTR in treating GI-NENLM by analyzing data from the Surveillance, Epidemiology, and End Results Program (SEER) and the First Affiliated Hospital of Sun Yat-sen University (FAH).

**Methods:**

The SEER Registry 17 database and the FAH clinical pathology database were used to collect clinicopathology data for GI-NENLM diagnosed between 2010 and 2019 and between 2011 and 2022, respectively. Propensity score matching (PSM) was used to match the clinicopathological characteristics of patients from both cohorts. Inverse probability weighting (IPTW) was used to weigh the PTR and non-PTR groups. The primary endpoint was overall survival (OS).

**Results:**

After matching, 155 patients from the SEER database were matched to the FAH cohort. PTR was significantly associated with better prognosis in PSM-matched/unmatched SEER cohorts (*P* < 0.01) and in the FAH cohort even after eliminating selection bias using IPTW (*p* < 0.01). Subgroup analysis suggests that the cohort consisting of patients aged 55 years or older, individuals with colorectal primary tumors, those at the T1 disease stage, and those without extrahepatic metastasis may potentially benefit from PTR. Interaction analysis showed no significant interaction between PTR and other clinical and pathological factors except for age.

**Conclusion:**

The employment of PTR in patients with GI-NENLM is significantly correlated with individual survival benefits. We support performing PTR on carefully evaluated patients.

**Supplementary Information:**

The online version contains supplementary material available at 10.1186/s12876-023-03041-6.

## Introduction

Gastricintestinal Neuroendocrine neoplasms (GI-NENs) are a heterogeneous group of solid tumors that originate from the diffuse neuroendocrine cell system in the GI tract [1]. High genetic diversity of GI-NENs leads various peptide hormones and distinct hormonal syndromes. GI-NENs can be classified as functional or non-functional according to clinical symptoms [2]. Although GI-NENs share a low proportion in gastrointestinal neoplasm, there are still 6.9 newly diagnosis cases per 100,000 people [3]. And the morbidity of GI-NENs [4] keeps rising in recent years.

The prognosis of localized NENs have been proved more favorable with a longer median OS (> 30 years), compared to metastatic NENs (median OS: 12 months) [3, 5]. Unfortunately, approximately one-fifth of patients have distant metastases at their first diagnosis [6], and liver is the most commonly affected site, blamed for about 82% of metastasis cases [7]. Neuroendocrine neoplasm liver metastasis (NENLM) leads to worse survival rates, as most patients end up suffering liver failure and other tumor-relative complications [8]. Although there are several non-surgical treatment options for liver metastatic disease, surgery is an essential treatment and the only way to cure localized NENs, potentially increasing the quality of life and overall survival for most patients [9].

Several studies have investigated in the necessity and potential outcome improvement of PTR for patients with metastatic NENs [10–12]. A multicenter retrospective study including 854 patients, found that PTR in GI-NENs is associated with better survival, regardless of liver treatment or tumor grade [13]. Similarly, another study based on the SEER database found that PTR is an independent prognostic factor associated with prolonged overall survival in all patients with GI-NENLM [14].

In addition to the resection of the primary tumor, Surgical treatment for liver metastases plays an another significant role in the management of patients with GI-NENLM. Surgical options primarily involve liver resection (LR) and liver transplantation (LT). Current international guidelines recommend curative LR for well-differentiated NENLM in the absence of extrahepatic metastatic disease when feasible [1, 15]. LT is considered as a therapeutic option for selected unresectable NELM patients to achieve a curative approach while minimizing the risk of recurrence. However, the selection of patients for LT is a significant challenge, given the limited availability of donor pools in most countries. The role of LT in NELM remains a topic of debate.

Previous studies mainly focused on the European or North American population with limited clinical data, none has ever investigated the role of PTR in the Asian population. Furthermore, there have been very few studies exploring whether PTR interacts with clinical-pathological factors. Therefore, our study combined SEER and single-center GI-NENLM patient data from China, using matching and weighting methods to eliminate biases between the two databases as well as selection bias between the PTR and non-PTR groups, in order to investigate the effect of PTR on prognosis. Furthermore, we conducted subgroup analyses to explore the role of PTR in various subgroups and its interaction with important clinical and pathological factors.

## Materials and method

### SEER cohort selection

The data for this study were obtained by downloading the SEER 17 registries research database, which was extracted using the SEER*Stat version 8.4.1 software. Due to the utilization of anonymous data from the database, the requirement for institutional review board approval and individual patient consent was waived. The account 23,891-Nov2021 was authorized for access to search the SEER database. In summary, this study adhered to the principles outlined in the Declaration of Helsinki.

Data regarding patients with GI-NETLM were obtained from the SEER database. Inclusion criteria were as follows: [1] diagnosed between 2010 and 2019 [2]; the site recode “rare tumors” limited to ‘54 NET GEP’; and [3] selecting ‘YES’ for ‘SEER Combined Mets at DX-liver’ under the Extent of Disease category, and [4] selecting ‘10–98’ for ‘RX Summ-Surg Prim Site’ under the Therapy category. The exclusion criteria were as follows: [1] primary sites not originating in the stomach, small intestine (duodenum, jejunum, or ileum), or colorectum [2]; overall survival of fewer than 3 months; and [3] unknown survival time or censorship. For analytical and matching purposes, T staging was stratified into T1, T2, T3, and T4 categories; age was stratified at 55 years; the primary site was categorized as the stomach, small intestine, or colon; and the maximum diameter of the primary site was stratified at 2 cm. The ‘Undifferentiated carcinoma’ category in the ‘Grade’ was reclassified as NEC according to the WHO 2019 classification [16].

### FAH cohort selection

We retrospectively reviewed all patients from 2011 to 2022 at the First Affiliated Hospital of Sun Yat-sen University and conducted regular follow-ups for eligible patients. The period from diagnosis to all-cause death was referred to as OS. The inclusion criteria were as follows: [1] pathologically confirmed NET diagnosis, [2] clear pathological or radiological evidence of liver metastases, [3] the presence of liver metastases at the time of diagnosis, and [4] accurate information on primary lesion treatment. The exclusion criteria were as follows: [1] NET originating outside the gastrointestinal tract, [2] the absence of liver metastases preoperatively but detected in follow-up visit, [3] survival time less than 3 months or loss of clinical information. T staging was based on the seventh edition of the AJCC 7th classification, and grading was based on the 2019 WHO classification. The size of the primary site was measured postoperatively pathologically or with precise imaging.

In addition, we have collected more comprehensive information and patient treatment data for the FAH cohort, which facilitates the analysis of single-center data. For further details, please refer to Supplementary Table 1.

### Analysis of data

Baseline characteristics of the study population were compared using Pearson’s chi-square test, Fisher’s exact test, Student’s t-test, or the Mann-Whitney test. Categorical variables were presented as counts and percentages. Time-to-event data were estimated using the Kaplan-Meier method and compared using the log-rank test. Univariate Cox regression was used to explore potential factors that may affect prognosis, while stepwise regression was utilized to select variables for inclusion in the multivariate risk model.

Propensity score matching (PSM) analysis was performed at a 1:1 ratio to reduce potential bias between the SEER and FAH cohorts. The factors matched by PSM included: [1] age, [2] gender, [3] primary site, [4] grade, [5] T stage, [6] whether the patient underwent PTR, [7] size of the primary tumor and [8] presence of extrahepatic metastasis. Matched pairs were then formed using “nearest-neighbor” methods, with a caliper width of 0.05. Many clinical and pathological factors in the FAH cohort were unable to be matched with cases in the SEER cohort due to the insufficient information of SEER database and then were excluded from the matching process. However, we performed univariate and multivariate analyses of all potential prognostic factors, including those that were unmatched, to identify any factors that might affect prognosis. These analyses are presented primarily in Supplementary Table 2.

To adjust for the imbalance between patients who received and did not receive PTR, the inverse probability of treatment weighting (IPTW) was calculated based on the propensity score, which is defined as the inverse probability of patients receiving the treatment they actually received. The variables included in the propensity score were [1] age, [2] gender, [3] primary site, [4] grade, [5] T stage, [6] size of the primary tumor, and [7] presence of extrahepatic metastasis, To evaluate the degree of balance achieved after PSM and IPTW, we calculated standardized mean differences (SMDs) between the treatment groups. A SMD of ≤0.1 for each variable was considered an acceptable level of balance. IPTW weighting was performed on the SEER dataset before PSM matching, the SEER dataset after PSM matching, and the FAH dataset.

Univariate and multivariate Cox regression can be applied to all cohorts, irrespective of whether PSM or IPTW has been performed. The final analysis included a subgroup analysis based on clinical and pathological factors using univariate Cox regression, and forest plots of hazard ratio (HR) and confidence interval (CI) were generated for the PSM-matched SEER and FAH cohorts. Multiplicative interactions were examined to explore the interaction between PTR and each subgroup. When calculating the interaction, PTR*Subgroup was the main factor in each subgroup, with all other included subgroup variables as covariates, and the interaction *P* value was calculated.

All calculations and plotting were performed using R version 4.2.0, IBM SPSS Stat 26, Microsoft Excel 22, Adobe Acrobat 22, SEER*Stat version 8.4.1, and Adobe Photoshop 19.1.7. A *p*-value less than 0.05 was considered statistically significant.

## Results

### Patient characteristics of FAH cohort

**Fig. 1:**
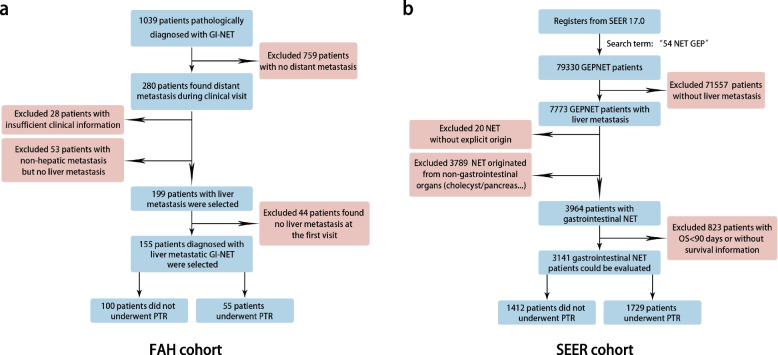
Patient-selection flowchart of FAH cohort (**a**) and SEER cohort (**b**)

1039 patients were diagnosed with GI-NENs at the First Affiliated Hospital of Sun Yat-sen University (FAH-SYSU) between 2011 and 2021. Of these patients, we excluded 759 localized GI-NENs cases and 28 cases without sufficient clinical data. An additional 53 patients were excluded for no liver metastasis and 44 patients were excluded whose liver metastases were not detected when diagnosed with NENs. Our study included 155 patients with GI-NENLM, among whom 55 (35.5%) received PTR. The primary tumors of 31 patients (31%) originated from the stomach, 33 patients (21.3%) from the small intestine, and 91 patients (58.7%) from the colorectum. The flowcharts were shown in Fig. 1a. Demographic data and clinicopathological factors of patients with GI-NENLM in FAH were listed and compared across the Resection and Non-resection Groups in Supplementary Table 1.

In the FAH cohort, Patients who underwent PTR tended to have negative CK levels (9% vs 27.3%, *p* = 0.005), and their NSE levels tended to be normal (34% vs 52.7%, *p* = 0.035). In terms of liver treatment, PTR was more likely to be performed in conjunction with the liver operation (4% vs 29.1%, *p* < 0.001). The above results are shown in Supplementary Table 1.

### SEER cohort and propensity score matching (PSM)

A total of 3141 registered patients of the SEER database were enrolled for analysis, including 1792 (57.1%) patients accepting primary tumor resection (PTR) and 1412 patients not accepting PTR (32.9%). The male-to-female ratio was 1.09:1 among the cohort. The most common original tumor site was the small intestine (2119, 67.5%) followed by the colorectum (705, 22.4%) and stomach (317, 10.1%). Among 1797 patients with specific tumor differentiation, 901 cases are well differentiation (G1) lesions, 348 cases are moderate differentiation (G2) lesions, 352 cases are poor differentiation lesions and 196 patients suffered neuroendocrine cancer (NEC). 411 (13.1%) patients had extrahepatic metastases, which were not found in 2730 (86.9%) patients. The flowchart were shown in Fig. 1b.

There was a significant difference in most of the patient characteristics (age, primary, differentiation, T classification, primary tumor resection, extrahepatic metastases, Table 1.) between the SEER cohort and FAH cohort. For the purpose of Enhancing comparability, we exert a 1:1 PSM on the SEER cohort based on clinicopathologic characteristics. Patients with complete clinicopathological information from the SEER cohort were included in PSM, and 155 well-matched patients were selected for further analysis. After PSM, all characteristics were balanced except differentiation (Table 1).

**Table 1:** Patients’ baselines before and after PSM Matched

Characteristics	Before PSM				After PSM			
	SEER	FAH	SMD	*P*-value	SEER	FAH	SMD	*P*-value
	(*N* = 3141)	(*N* = 155)			(*N* = 155)	(*N* = 155)		
Age			−0.110	< 0.001			0.013	0.909
< 55	863 (27.5%)	67 (43.2%)			69 (44.5%)	67 (43.2%)		
> =55	2278 (72.5%)	88 (56.8%)			86 (55.5%)	88 (56.8%)		
Sex			0.079	0.18			0.000	1
Female	1501 (47.8%)	65 (41.9%)			65 (41.9%)	65 (41.9%)		
Male	1640 (52.2%)	90 (58.1%)			90 (58.1%)	90 (58.1%)		
PTS				< 0.001				0.151
Stamoch	317 (10.1%)	31 (20.0%)	0.146		38 (24.5%)	31 (20.0%)	−0.045	
Small intestine	2119 (67.5%)	33 (21.3%)	−0.540		43 (27.7%)	33 (21.3%)	−0.065	
Colorectum	705 (22.4%)	91 (58.7%)	0.393		74 (47.7%)	91 (58.7%)	0.110	
Grade				< 0.001				< 0.001
Well differentiation G1	901 (28.7%)	21 (13.5%)	− 0.415		31 (20.0%)	21 (13.5%)	−0.065	
Moderate differentiation G2	348 (11.1%)	112 (72.3%)	0.529		57 (36.8%)	112 (72.3%)	0.355	
Poor differentiation G3	352 (11.2%)	12 (7.7%)	−0.080		40 (25.8%)	12 (7.7%)	−0.181	
NEC	196 (6.2%)	10 (6.5%)	−0.034		27 (17.4%)	10 (6.5%)	−0.110	
Missing	1344 (42.8%)	0 (0%)						
T				< 0.001				0.66
T1	43 (1.4%)	29 (18.7%)	0.154		21 (13.5%)	29 (18.7%)	0.052	
T2	188 (6.0%)	57 (36.8%)	0.214		60 (38.7%)	57 (36.8%)	−0.019	
T3	559 (17.8%)	36 (23.2%)	−0.218		37 (23.9%)	36 (23.2%)	−0.007	
T4	434 (13.8%)	33 (21.3%)	−0.150		37 (23.9%)	33 (21.3%)	−0.026	
Missing	1917 (61.0%)	0 (0%)						
PTR			−0.514	< 0.001			−0.065	0.294
No	1412 (45.0%)	100 (64.5%)			90 (58.1%)	100 (64.5%)		
Yes	1729 (55.0%)	55 (35.5%)			65 (41.9%)	55 (35.5%)		
Primary tumor size			0.011	0.901			−0.013	0.895
< 2	307 (9.8%)	39 (25.2%)			37 (23.9%)	39 (25.2%)		
> =2	954 (30.4%)	116 (74.8%)			118 (76.1%)	116 (74.8%)		
Missing	1880 (59.9%)	0 (0%)						
Extrahepatic metastases			0.253	< 0.001			0.065	0.263
No	2730 (86.9%)	104 (67.1%)			114 (73.5%)	104 (67.1%)		
Yes	411 (13.1%)	51 (32.9%)			41 (26.5%)	51 (32.9%)		

*PTR* primary tumor resection, *PTS* primary tumor site, *NEC* neuroendocrine carcinoma, *HR* hazard ratio, *CI* confidence interval, *PSM* Propensity matching analysis, *SMD* Standard Mean Difference

### Overall survival of patients with or without PTR

**Fig. 2:**
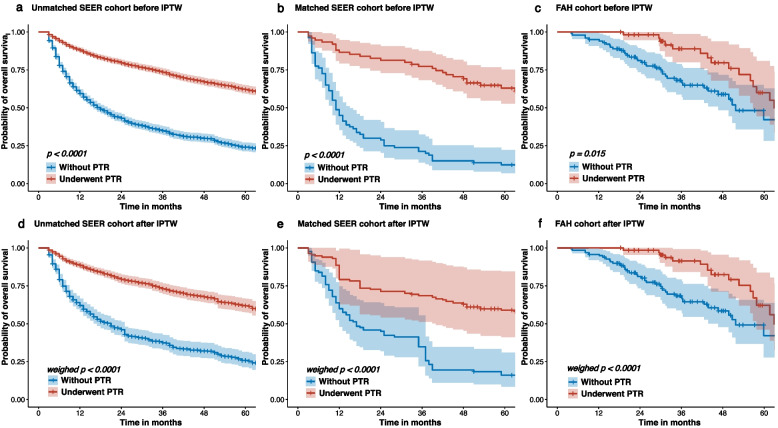
Overall survival of patients with and without PTR. **a** Overall survival of pre-PSM SEER cohort before IPTW stratified by with and without PTR. **b** Overall survival of post-PSM SEER cohort before IPTW stratified by with and without PTR. **c** Overall survival of FAH cohort before IPTW stratified by with and without PTR. **d** Overall survival of pre-PSM SEER cohort after IPTW stratified by with and without PTR. **e** Overall survival of post-PSM SEER cohort after IPTW stratified by with and without PTR. **f** Overall survival of FAH cohort after IPTW stratified by with and without PTR

In the pre-matched SEER cohort before performing IPTW, PSM-matched SEER cohort and FAH cohort without IPTW weighting, patients who underwent PTR had a significantly longer survival time compared to those who did not undergo PTR (pre-matched SEER cohort, median survival: 18 months vs 100 months, *p* < 0.001, Fig. 2a; post-matched SEER cohort, median survival: 11 months vs 81 months, *p* < 0.001, Fig. 2b; FAH cohort, median survival: 51.7 months vs 62.9 months, *p* < 0.001, Fig. 2c). Similarly, In the pre-matched SEER cohort after performing IPTW weighting and in the PSM-matched SEER cohort and unmatched FAH cohort with IPTW weighting, patients who underwent PTR still had a significantly better survival time compared to those who did not undergo PTR in the pre-matched SEER cohort (median survival: 21 months vs 93 months, weighted *p* < 0.001, Fig. 2d), post-matched SEER cohort (median survival: 15 months vs 51 months, weighted *p* < 0.001, Fig. 2e), and FAH cohort (median survival: 51.7 months vs 62.9 months, weighted *p* < 0.001, Fig. 2f).

### Inverse probability of treatment weighting (IPTW) and survival analysis

To minimize the selection bias, we exerted IPTW on the pre-PSM SEER cohort, post-PSM SEER cohort, and FAH cohort. Before implementing IPTW for the PTR group and non-PTR group, the baseline was not aligned in all cohorts except for the FAH cohort. After IPTW, the baselines of all cohorts in both PTR and non-PTR groups became comparable, with a significant increase in comparability (all SMDS decreased, see Tables 1, 2 and 3; Supplementary Table 3).

**Table 2:** Comparison of baseline characteristics in a post-PSM SEER cohort before and after IPTW adjustment

Characteristics	Before IPTW				After IPTW			
	Without PTR	PTR	*P*-value	SMD	Without PTR	PTR	*P*-value	SMD
	(*N* = 90)	(*N* = 65)			(*N* = 139.1)	(*N* = 149.0)		
Age			0.0684	−0.1607			0.611	0.0493
< 55	34 (37.8%)	35 (53.8%)			54.8 (39.4%)	51.0 (34.3%)		
> =55	56 (62.2%)	30 (46.2%)			84.4 (60.6%)	98.0 (65.7%)		
Sex			0.562	0.0598			0.910	0.0163
Female	40 (44.4%)	25 (38.5%)			60.9 (43.8%)	63.3 (42.4%)		
Male	50 (55.6%)	40 (61.5%)			78.2 (56.2%)	85.8 (57.6%)		
PTS			< 0.001				0.579	
Stamoch	27 (30.0%)	11 (16.9%)		−0.1308	37.9 (27.3)	38.8 (26.1)		− 0.0049
Small intestine	10 (11.1%)	33 (50.8%)		0.3966	26.0 (18.7)	43.0 (28.9)		0.0913
Colorectum	53 (58.9%)	21 (32.3%)		−0.2658	75.2 (54.1)	67.1 (45.1)		−0.0864
Grade			< 0.001				0.803	
Well differentiation G1	17 (18.9%)	14 (21.5%)		0.0265	30.1 (21.6)	29.3 (19.7)		−0.0207
Moderate differentiation G2	12 (13.3%)	45 (69.2%)		0.559	40.4 (29.0)	57.1 (38.3)		0.0947
Poor differentiation G3	36 (40.0%)	4 (6.2%)		−0.3385	41.7 (30.0)	43.7 (29.3)		−0.0072
NEC	25 (27.8%)	2 (3.1%)		−0.247	27.0 (19.4)	18.8 (12.6)		−0.0667
T			0.252				0.638	
T1	12 (13.3%)	9 (13.8%)		0.0051	22.4 (16.1)	15.0 (10.1)		−0.0603
T2	40 (44.4%)	20 (30.8%)		−0.1368	54.2 (39.0)	48.1 (32.3)		−0.0643
T3	17 (18.9%)	20 (30.8%)		0.1188	27.3 (19.6)	43.9 (29.5)		0.0962
T4	21 (23.3%)	16 (24.6%)		0.0128	35.2 (25.3)	42.0 (28.2)		0.0284
Primary tumor size			< 0.001	−0.3573			0.293	
< 2	8 (8.9%)	29 (44.6%)			21.4 (15.4%)	35.8 (24.0%)		−0.087
> =2	82 (91.1%)	36 (55.4%)			117.7 (84.6%)	113.2 (76.0%)		
Extrahepatic metastases			0.0135	−0.1906			0.449	−0.0865
No	59 (65.6%)	55 (84.6%)			99.9 (71.8%)	119.9 (80.4%)		
Yes	31 (34.4%)	10 (15.4%)			39.2 (28.2%)	29.1 (19.6%)		

*PTR* primary tumor resection, *PTS* primary tumor site, *NEC* neuroendocrine carcinoma, *HR* hazard ratio, *CI* confidence interval, *SMD* standard mean difference, *PSM* propensity matching analysis, *IPTW* inverse probability of treatment weighting, *SMD* Standard Mean Difference

**Table 3:** Comparison of baseline characteristics in FAH cohort before and after IPTW adjustment

Characteristics	Before IPTW				After IPTW			
	Without PTR	PTR	*P*-value	SMD	Without PTR	PTR	*P*-value	SMD
	(*N* = 100)	(*N* = 55)			(*N* = 155.0)	(*N* = 154.1)		
Age			0.806	−0.0345			0.902	−0.012
< 55	42 (42.0%)	25 (45.5%)			67.6 (43.6%)	68.9 (44.7%)		
> =55	58 (58.0%)	30 (54.5%)			87.4 (56.4%)	85.2 (55.3%)	0.846	
Sex			0.882	−0.0264				−0.017
Female	41 (41.0%)	24 (43.6%)			64.2 (41.4%)	66.5 (43.1%)		
Male	59 (59.0%)	31 (56.4%)			90.8 (58.6%)	87.6 (56.9%)	0.951	
PTS			0.89					
Stamoch	19 (19.0%)	12 (21.8%)		0.0282	29.7 (19.2)	28.2 (18.3)		−0.011
Small intestine	21 (21.0%)	12 (21.8%)		0.0082	33.1 (21.3)	30.2 (19.6)		−0.011
Colorectum	60 (60.0%)	31 (56.4%)		−0.0364	92.3 (59.5)	95.7 (62.1)	0.998	0.022
Grade			0.535					
Well differentiation G1	14 (14.0%)	7 (12.7%)		−0.0127	20.3 (13.1)	19.5 (12.6)		−0.004
Moderate differentiation G2	70 (70.0%)	42 (76.4%)		0.0636	112.1 (72.3)	113.5 (73.6)		0.126
Poor differentiation G3	10 (10.0%)	2 (3.6%)		−0.0636	11.9 (7.7)	10.7 (6.9)		0.008
NEC	6 (6.0%)	4 (7.3%)		0.0127	10.6 (6.9)	10.5 (6.8)		0.000
T			0.37				0.989	
T1	16 (16.0%)	13 (23.6%)		0.0764	29.1 (18.8)	27.7 (18.0)		−0.007
T2	41 (41.0%)	16 (29.1%)		−0.1191	57.5 (37.1)	57.8 (37.5)		0.004
T3	21 (21.0%)	15 (27.3%)		0.0627	35.9 (23.2)	38.9 (25.3)		0.021
T4	22 (22.0%)	11 (20.0%)		−0.02	32.5 (21.0)	29.7 (19.3)		
Primary tumor size			0.0713	−0.1455			0.986	−0.001
< 2	20 (20.0%)	19 (34.5%)			39.1 (25.2%)	39.1 (25.4%)		
> =2	80 (80.0%)	36 (65.5%)			115.9 (74.8%)	115.0 (74.6%)		
Extrahepatic metastases			0.885	0.0255			0.826	0.018
No	68 (68.0%)	36 (65.5%)			101.8 (65.7%	98.3 (63.8%)		
Yes	32 (32.0%)	19 (34.5%)			53.2 (34.3%)	55.8 (36.2%)		

*PTR* primary tumor resection, *PTS* primary tumor site, *NEC* neuroendocrine carcinoma, *HR* hazard ratio, *CI* confidence interval, *SMD* standard mean difference, *PSM* propensity matching analysis, *IPTW* inverse probability of treatment weighting, *SMD* Standard Mean Difference

Then, we exerted the survival analysis on the above cohorts before and after IPTW. Univariate Cox regression analysis indicated a significant association between PTR and improved patient prognosis across all cohorts (all HRs < 1, *p* < 0.05, Tables 4 and 5; Supplementary Table 4.). The positive correlation between PTR and better patient outcomes remained independently significant in multivariate Cox analysis, irrespective of IPTW application. This finding demonstrated robust consistency in the FAH and SEER cohorts following PSM matching (All multivariate HRs < 1, multivariate *p* < 0.01; Tables 4 and 5; Supplementary Table 4.)

**Table 4:** Univariate and multivariate cox regression analysis before and after IPTW adjustment in post-PSM SEER cohort

	Before IPTW weighted				After IPTW weighted			
	Univariate cox regression		Multivariate cox regression		Univariate cox regression		Multivariate cox regression	
	HR (95%CI)	*P* value	HR (95%CI)	*P* value	HR (95%CI)	*P* value	HR (95%CI)	*P* value
Age								
< 55	Reference				Reference			
> =55	1.69 (1.15–2.48)	0.007			1.53 (0.95–2.45)	0.079		
Sex								
Female	Reference				Reference		Reference	
Male	1.06 (0.73–1.54)	0.778			1.28 (0.75–2.18)	0.373	1.53 (0.92–2.54)	0.100
PTS								
Stamoch	Reference		Reference		Reference		Reference	
Small intestine	0.21 (0.11–0.38)	< 0.001	0.51 (0.25–0.14)	0.064	0.26 (0.13–0.51)	< 0.001	0.49 (0.22–1.09)	0.079
Colorectum	0.98 (0.64–1.5)	0.931	1.16 (0.75–1.77)	0.507	0.85 (0.44–1.63)	0.614	0.90 (0.48–1.69)	0.747
Grade								
Well differentiation G1	Reference		Reference		Reference		Reference	
Moderate differentiation G2	0.98 (0.55–1.76)	0.951	1.59 (0.86–2.93)	0.141	1.46 (0.85–2.49)	0.167	1.60 (0.92–2.79)	0.098
Poor differentiation G3	5.78 (3.24–10.31)	< 0.001	4.11 (2.23–7.58)	< 0.001	2.87 (1.15–7.94)	0.024	2.86 (1.27–6.45)	0.011
NEC	5.40 (2.9–10.05)	< 0.001	2.81 (1.46–5.41)	0.002	6.68 (3.28–13.59)	< 0.001	4.12 (1.88–9.06)	< 0.001
T								
T1	Reference				Reference		Reference	
T2	1.48 (0.8–2.74)	0.214			1.60 (0.83–3.11)	0.162	0.49 (0.12–2.08)	0.337
T3	1.18 (0.61–2.23)	0.623			1.30 (0.21–3.31)	0.583	0.52 (0.13–2.08)	0.356
T4	1.76 (0.91–3.5)	0.090			2.89 (1.38–6.05)	0.005	0.73 (0.17–3.19)	0.679
PTR								
No	Reference		Reference		Reference		Reference	
Yes	0.24 (0.16–0.37)	< 0.001	0.40 (0.24–0.66)	< 0.001	0.41 (0.23–0.71)	< 0.001	0.39 (0.21–0.74)	0.004
Primary tumor size								
< 2	Reference		Reference		Reference		Reference	
> =2	4.02 (2.29–7.07)	< 0.001	1.78 (0.96–3.29)	0.066	2.79 (1.46–5.33)	0.002	2.07 (0.53–8.18)	0.297
Extrahepatic metastases								
No	Reference				Reference		Reference	
Yes	2.30 (1.54–3.43)	< 0.001			2.44 (1.49–3.98)	< 0.001	1.68 (0.93–3.01)	0.083

*PTR* primary tumor resection, *PTS* primary tumor site, *NEC* neuroendocrine carcinoma, *HR* hazard ratio, *CI* confidence interval, *PSM* propensity matching analysis, *IPTW*: inverse probability of treatment weighting;

**Table 5:** Univariate and multivariate cox regression analysis before and after IPTW adjustment in FAH cohort

	Before IPTW weighted				After IPTW weighted			
	Univariate cox regression		Multivariate cox regression		Univariate cox regression		Multivariate cox regression	
	HR (95%CI)	*P* value	HR (95%CI)	*P* value	HR (95%CI)	*P* value	HR (95%CI)	*P* value
Age								
< 55	Reference		Reference		Reference		Reference	
> =55	1.73 (0.98–3.06)	0.057	1.62 (0.87–3.05)	0.130	1.36 (0.75–2.46)	0.312	1.48 (0.77–2.83)	0.236
Sex								
Female	Reference				Reference			
Male	1.51 (0.87–2.62)	0.144			1.51 (0.83–2.77)	0.178		
PTS								
Stamoch	Reference				Reference		Reference	
Small intestine	0.32 (0.13–0.79)	0.013			0.37 (0.16–0.85)	0.019	0.39 (0.15–1.04)	0.059
Colorectum	0.54 (0.29–0.99)	0.048			0.44 (0.24–0.83)	0.011	0.38 (0.18–0.82)	0.014
Grade								
Well differentiation G1	Reference		Reference		Reference		Reference	
Moderate differentiation G2	1.40 (0.55–3.6)	0.483	1.42 (0.53–3.78)	0.483	1.35 (0.6–3.03)	0.466	1.57 (0.66–3.72)	0.306
Poor differentiation G3	3.38 (1.12–10.22)	0.031	2.54 (0.77–8.34)	0.124	1.52 (0.35–6.65)	0.576	1.02 (0.3–3.47)	0.973
NEC	6.08 (3.6–18.3)	0.001	5.11 (1.51–17.31)	0.009	4.96 (1.53–16.1)	0.008	3.86 (1.1–13.62)	0.036
T								
T1	Reference				Reference			
T2	0.83 (0.35–2)	0.685			0.72 (0.3–1.73)	0.456		
T3	1.46 (0.64–3.32)	0.363			1.07 (0.42–2.7)	0.886		
T4	2.13 (0.95–4.78)	0.066			1.84 (0.86–3.94)	0.117		
PTR								
No	Reference		Reference		Reference		Reference	
Yes	0.49 (0.27–0.88)	0.018	0.29 (0.14–0.59)	< 0.001	0.44 (0.25–0.77)	0.005	0.26 (0.13–0.52)	< 0.001
Primary tumor size								
< 2	Reference		Reference		Reference		Reference	
> =2	3.17 (1.35–7.42)	0.008	2.23 (0.93–5.38)	0.073	2.51 (1.05–5.96)	0.038	1.98 (0.82–4.81)	0.131
Extrahepatic metastases								
No	Reference		Reference		Reference		Reference	
Yes	2.5 (1.46–4.28)	< 0.001	2.84 (1.58–5.11)	< 0.001	2.73 (1.55–4.8)	< 0.001	3.35 (1.67–6.72)	< 0.001

*PTR* primary tumor resection, *PTS* primary tumor site, *NEC* neuroendocrine carcinoma, *HR* hazard ratio, *CI* confidence interval, *PSM* propensity matching analysis, *IPTW* inverse probability of treatment weighting

Besides, we found in the above analysis that tumor differentiation is another prognostic factor, which was highly accordant in three cohorts before or after IPTW (Supplementary Table 3, Tables 4 and 5). Poor differentiation tumors were related to a higher HR, and this conclusion was consistent in both univariate and multivariate Cox regression.

### Subgroup and intersection analysis of patients with GI-NENLM

Subgroup analyses on matched cohorts were conducted from the SEER and FAH databases based on [1] age, [2] gender, [3] primary site, [4] grade, [5] T stage, [6] primary tumor size, and [7] extrahepatic metastasis to investigate the effect of PTR in each subgroup. The patient numbers and distribution in each subgroup are presented in Table 1.

The forest plot of the subgroup analysis is presented in Table 6. As shown in the table, consistent results were observed in the subgroup analysis of PSM-matched SEER and FAH subgroups. Specifically, PTR was significantly associated with better prognosis in the subgroups of patients aged 55 and over (SEER cohort, HR: 0.32, 95%CI: 0.19–0.55, *p* < 0.001; FAH cohort, HR: 0.31, 95%CI: 0.14–0.69, *p* = 0.004), primary tumor located in the colorectum (SEER cohort, HR: 0.34, 95%CI: 0.18–0.64, *p* < 0.001; FAH cohort, HR: 0.27, 95%CI: 0.11–0.65, *p* = 0.003), T1 stage (SEER cohort, HR: 0.31, 95%CI: 0.1–0.98, *p* = 0.046; FAH cohort, HR: 0.1, 95%CI: 0.01–0.85, *p* = 0.034, and presence of extrahepatic metastasis (SEER cohort, HR: 0.15, 95%CI: 0.04–0.51, *p* = 0.003; FAH cohort, HR: 0.21, 95%CI: 0.07–0.58, *p* = 0.003).

**Table 6:**
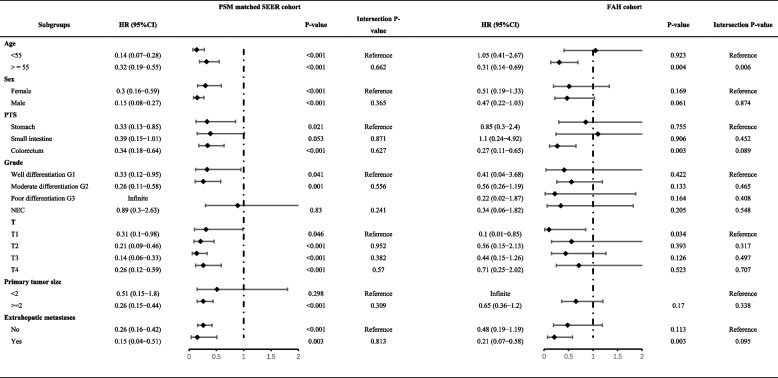
The role of PTR and interaction analysis across subgroups

Furthermore, we performed interaction analyses in matched SEER and FAH cohorts stratified by different subgroups. The results showed that in the SEER cohort, there were no significant interactions between PTR and any of the studied factors in terms of survival outcomes (all interaction *p*-values > 0.2, Table 6). Furthermore, in the FAH cohort, except for age, PTR did not interact significantly with other factors on survival (all intersection *p*-values > 0.05, Table 6). Regarding the interaction between age and PTR, we found a positive interaction between age > = 55 years and PTR (HR: 6.19, 95%CI: 1.67–23.00, *p* = 0.006).

## Discussion

The NCCN guidelines recommend PTR for certain cases of NENs, especifically those with local complications such as GI tract stricture, bleeding, or perforation. However, the role of PTR for advanced NET patients without complications remains uncertain [17]. The ESMO Clinical Practice Guidelines for GEP-NENs indicate that surgery may be appropriate for selected patients based on elaborate evaluation of tumor grading, LMs distribution, and primary site [1]. Above all, the criteria of targeted patients for PTR remain a complicated and pending issue.

Several retrospective studies have reported improved survival in GI-NET patients with distant metastasis who underwent PTR [13, 18, 19], while the conflicting result was concluded by certain research [20]. The UKINETs study was based on the European population including 380 midgut-originating GI-NENLM patients, which demonstrated that PTR (*P* = 0.015) was an independent predictor for better survival [21]. Many previous studies have found that PTR is more suitable for certain specific populations. For instance, studies by Selberherr et al. [19] and guidelines from the (ENETS) [22] have suggested that PTR is a viable option for patients with small bowel NET and distant metastasis. Citterio, D. et al’s research also supports the use of PTR in small bowel NENs, as it reduces the risk of local complications such as intestinal obstruction, perforation, and bleeding [23]. Similarly, For pancreatic neuroendocrine tumors (Pan-NETs), the ESMO guidelines state that patients with highly functional Pan-NETs with a high tumor burden may benefit from tumor debulking surgery (e.g., insulinomas, vasoactive intestinal peptide (VIP)omas), and surgery is typically recommended for this indication. There is debate over whether palliative resection of non-functional Pan-NETs is necessary since the risk of tumor-related symptoms is low [1]. The study conducted by Yoshida et al. also suggested that surgery did not lead to improved survival in patients with advanced pancreatic NEC [24]. In cases of NEN with an unknown primary tumor origin, the primary consideration is to search for the primary site because treatment of the primary tumor, especially resection of the primary tumor, can significantly enhance the survival rate [25]. On the other hand, Alexandra et al. [26] found that patients with early T staging may benefit more from PTR. Wheras poorly differentiated NECs typically have worse survival, even than those of adenocarcinoma [27]. Surgery is not a routine decision for patients with poorly differentiated NECs. Other clinicopathological factors, including tumor size, and the situation of liver metastases, have been established as important prognostic factors for GI-NENLM in several studies [28–30]. In summary, controversy persists over whether a patient with GINENLM should undergo PTR, with different institutions and individual physicians making varying decisions.

Previous research, whether based on the SEER database or large-scale single or multi-center studies, has primarily targeted Western populations. To date, no studies have been conducted specifically on Asian populations. While it is widely recognized that the clinical symptoms, biological behavior, and prognosis of NETs are closely associated with factors such as the primary site of origin, functional status, hormone secretion, differentiation, and complications, which may have limited relevance to ethnic distribution [31–33], studies have indicated differences in the incidence of NETs between Asian and Western populations. For instance, the most common primary site of GEP-NETs in Asian populations is the rectum, whereas in European and North American populations [34], GEP-NETs are most commonly found in the small intestine [35]. This observation is further supported by our study, in which 58.7% of cases originated in the colorectal region, aligning with this pattern. Therefore, the absence of research on PTR in Asian patients may raise doubts about the universality of PTR benefits. Furthermore, most studies have not matched patients’ baseline characteristics, resulting in uneven group baselines for PTR and non-PTR cohorts, which may introduce selection bias and compromise the reliability of the findings. To broaden the potential benefits of PTR and further enhance its support base, it is crucial to conduct research involving diverse populations and ensure a rigorous study design that accounts for baseline characteristics and minimizes selection bias.

Therefore, our study, for the first time, includes data on PTR in Asian populations and records additional clinical and pathological factors such as Ki67 and tumor markers. We utilized PSM to match the SEER database with our single-center data, thus increasing the comparability of results across different populations. Additionally, we employed IPTW to minimize potential selection bias, thus obtaining more generalizable and reliable conclusions about the effect of PTR on patient prognosis. Furthermore, we analyzed interactions to gain a more detailed understanding of the role of PTR in patient outcomes. To our knowledge, this is the first study that has matched the SEER database with an Asian population to investigate the role of PTR in GI-NENLM.

In our study, we matched the FAH and SEER cohorts to achieve well-balanced baselines and found that PTR was independently associated with improved prognosis in patients. This suggests that, among our selected cohort, PTR may also be related to patient outcomes, supporting the use of PTR for GI-NENLM. The conclusions remained consistent after using IPTW to remove as much selection bias as possible. By rigorously controlling for potential biases, our study presents a higher level of confidence in these findings.

Additionally, we performed subgroup analysis and interaction analysis for the FAH and SEER cohort. The consensus is that PTR may benefit in patients with age > = 55 years, primary tumor in the colorectum T1 stage, and presence of extrahepatic metastasis. The interaction analysis revealed that except for age, PTR had no interaction with other factors (all intersection-*P* > 0.05, Table 6). This suggests that in the FAH subgroups, PTR may independently affect the prognosis apart from other factors, except age. In this study, we found that performing PTR had a positive interaction with the age > = 55 years old (HR: 6.19, 95%CI: 1.67–23.00, *p* = 0.006). The impact of surgery on prognosis may be more significant in age 55 or older patients as other health issues may overshadow the effect of surgery. The potential role of increased surgical intervention in patients aged 55 or older remains to be further explored in future studies.

Upon comparing the matched FAH and SEER cohorts using subgroup analyses, we found that PTR was significantly associated with better prognosis in subgroups characterized by advanced age (> = 55), primary tumors located in the colorectum, shallow invasion depth (T1), and the presence of extrahepatic metastasis. The inconsistent results in the subgroup analysis could be attributed to the relatively small sample size and the large number of subgroups analyzed (19 in total). Moreover, despite the PSM matching of the two groups, the baseline characteristics were not completely balanced, and there may still be potential selection bias that was not accounted for in the analysis. Despite this, we found consistency in our analysis of two different populations, strongly suggesting that PTR may be independently associated with better prognosis in certain patients with GI-NENLM.

Additionally, despite the stringent criteria and contentious patient selection for LR and LT, there remains a compelling rationale for and discernible benefits of primary tumor resection in the context of liver metastatic sites. Yves Patrice Le Treut et al. [36] conducted a study involving 85 liver transplant patients and found that patients who underwent PTR before LT exhibited significantly improved survival compared to those who did not (PTR: > 60 months vs. NoPTR: 26 months). Studies conducted by Rajeev Dhupar et al. [37] highlighted the potential survival benefits of simultaneous PTR and LT for patients with NENLM. In their research involving 2320 GEP-NET patients from the National Cancer Database (NCDB) [38], Qichen Chen et al. made a noteworthy discovery: patients undergoing both PTR and LR experienced the most substantial survival advantage compared to those subjected to other surgical interventions (*P* < 0.001). In summary, PTR may exert an underlying influence that enhances patients’ prognoses for subsequent treatments at the metastatic site. Given the limited sample size in our study, which precluded the inclusion of a sufficient number of patients undergoing both PTR and LR, future investigations on a larger scale are warranted to delve deeper into the relationship between PTR and the metastatic site.

Numerous factors can significantly impact GI-NENLM prognoses, such as the Ki67 proliferation index [39, 40], liver tumor load [41], and surgical interventions [42, 43]. Our study identified the Ki67 index as an independent prognostic factor for GI-NENLM patients (MVHR: 4.09, 95%CI: 2.16–7.76, *p* < 0.001) and found that TACE/TAE treatments were associated with better survival outcomes (HR: 0.36, 95% CI: 0.21–0.62, p < 0.001), consistent with findings from Touloupas et al. [44] and Fiore et al. [45] studies. However, due to the SEER database’s limitations, we could only analyze the impact of these factors using our single-center data. Further research with larger sample sizes is needed to investigate these factors more comprehensively.

Our study has limitations. First, we utilized the SEER database to match samples from a single Chinese center, potentially limiting representativeness. Second, despite employing PSM and IPTW to minimize bias, uncontrolled factors such as liver metastasis burden, postoperative treatment, and liver therapy may introduce selection biases. Despite these limitations, our study strongly supports PTR’s potential role in GI-NENLM patients by corroborating SEER and FAH data. Further large-scale prospective studies are warranted to explore PTR’s role in GI-NENLM patients more thoroughly.

## Conclusion

Our study, with rigorous bias control, further supports the positive role of PTR in treating GI-NENLM and expands the potential applicability of this treatment modality. Our findings endorse the use of PTR surgery in specific GI-NENLM patient populations.

### Supplementary Information

**Additional file 1.**

## Data Availability

The datasets analyzed during the current study are available in the Clinical Pathology Database of the First Affiliated Hospital of Sun Yat-Sen University and the SEER Program (www.seer.cancer.gov). SEER*Stat Database: Incidence- SEER 17 Regs Research Data, Nov 2019 Sub (2000–2019 varying).
