# Supplementary material for: Resection of the primary tumor improves the prognosis of gastrointestinal neuroendocrine neoplasms with liver metastases: mutual validation based on SEER database and institutional data
Source: BMC Gastroenterol. 2023 Nov 23;23:408. doi: 10.1186/s12876-023-03041-6 (PMC10666352; doi:10.1186/s12876-023-03041-6)
Supplement: Supplementary file 1 — Additional file 1. [file 12876_2023_3041_MOESM1_ESM.docx]

| **Supplementary Table1. Full Demographics and Comparison of Primary Tumor Resection and Non-resection Groups for FAH Cohort** | | | | |  |
| --- | --- | --- | --- | --- | --- |
|  |  |  |  |  |  |
|  | **Total** | **No PTR (N=100)** | **PTR (N=55)** | **P value** |  |
|  |  |  |  |  |  |
| **Sex** |  |  |  | 0.882 |  |
| Female | 65 (41.9%) | 41 (41.0%) | 24 (43.6%) |  |  |
| Male | 90 (58.1%) | 59 (59.0%) | 31 (56.4%) |  |  |
| **Age(year)** |  |  |  | 0.291 |  |
| Mean (SD) | 53.7 (11.2) | 54.4 (11.0) | 52.3 (11.6) |  |  |
| Median [Min, Max] | 56.0 [15.0, 75.0] | 56.0 [15.0, 75.0] | 55.0 [26.0, 73.0] |  |  |
| **Primary tumor site** |  |  |  | 0.890 |  |
| Stomach | 31 (20.0%) | 19 (19.0%) | 12 (21.8%) |  |  |
| Small Intestinal | 33 (21.3%) | 21 (21.0%) | 12 (21.8%) |  |  |
| Colorectum | 91 (58.7%) | 60 (60.0%) | 31 (56.4%) |  |  |
| **LM** |  |  |  | 0.981 |  |
| No | 21 (13.5%) | 13 (13.0%) | 8 (14.5%) |  |  |
| Yes | 134 (86.5%) | 87 (87.0%) | 47 (85.5%) |  |  |
| **T stage** |  |  |  | 0.370 |  |
| 1 | 29 (18.7%) | 16 (16.0%) | 13 (23.6%) |  |  |
| 2 | 57 (36.8%) | 41 (41.0%) | 16 (29.1%) |  |  |
| 3 | 36 (23.2%) | 21 (21.0%) | 15 (27.3%) |  |  |
| 4 | 33 (21.3%) | 22 (22.0%) | 11 (20.0%) |  |  |
| **Ki67(%)** |  |  |  | 0.410 |  |
| ≤ 30 | 138(89%) | 87 (87.0%) | 51 (92.7%) |  |  |
| > 30 | 17(11%) | 13 (13.0%) | 4 (7.3%) |  |  |
| **Grade** |  |  |  | 0.568 |  |
| NET-G1 | 21 (13.5%) | 14 (14.0%) | 7 (12.7%) |  |  |
| NET-G2 | 112 (72.3%) | 70 (70.0%) | 42 (76.4%) |  |  |
| NET-G3 | 12 (7.1%) | 10 (10.0%) | 2 (3.6%) |  |  |
| NEC | 10 (6.5%) | 6 (6.0%) | 4 (7.3%) |  |  |
| **Primary tumor size** |  |  |  | 0.0713 |  |
| <2 | 39 (25.2%) | 20 (20.0%) | 19 (34.5%) |  |  |
| >=2 | 116 (74.8%) | 80 (80.0%) | 36 (65.5%) |  |  |
| **Extrahepatic metastases** | |  |  | 0.885 |  |
| No | 104 (67.1%) | 68 (68.0%) | 36 (65.5%) |  |  |
| Yes | 51 (32.9%) | 32 (32.0%) | 19 (34.5%) |  |  |
| **Liver tumor size(cm)** |  |  |  | 0.094 |  |
| <5 | 84 (54.2%) | 48 (48.0%) | 36 (65.5%) |  |  |
| 5~10 | 52 (33.5%) | 37 (37.0%) | 15 (27.3%) |  |  |
| >10 | 19 (12.3%) | 15 (15.0%) | 4 (7.3%) |  |  |
| **Therapy of Liver** |  |  |  |  |  |
| **TAEorTACE** |  |  |  | 0.708 |  |
| No | 58 (37.4%) | 39 (39.0%) | 19 (34.5%) |  |  |
| Yes | 97 (62.6%) | 61 (61.0%) | 36 (65.5%) |  |  |
| **LO** |  |  |  |  |  |
| No | 135 (87.1%) | 96 (96.0%) | 39 (70.9%) | **<0.001** |  |
| Yes | 20 (12.9%) | 4 (4.0%) | 16 (29.1%) |  |  |
| **CD56** |  |  |  | 0.901 |  |
| Negative | 19 (12.3%) | 13 (13.0%) | 6 (10.9%) |  |  |
| Positive | 136 (87.7%) | 87 (87.0%) | 49 (89.1%) |  |  |
| **CgA** |  |  |  | 0.479 |  |
| Negative | 55 (35.5%) | 38 (38.0%) | 17 (30.9%) |  |  |
| Positive | 100 (64.5%) | 62 (62.0%) | 38 (69.1%) |  |  |
| **CK** |  |  |  | **0.005** |  |
| Negative | 24 (15.5%) | 9 (9.0%) | 15 (27.3%) |  |  |
| Positive | 131 (84.5%) | 91 (91.0%) | 40 (72.7%) |  |  |
| **NSE** |  |  |  | **0.036** |  |
| N | 63 (40.6%) | 34 (34.0%) | 29 (52.7%) |  |  |
| Over UNL | 92 (59.4%) | 66 (66.0%) | 26 (47.3%) |  |  |
| **CEA** |  |  |  | 0.765 |  |
| N | 135 (87.1%) | 86 (86.0%) | 49 (89.1%) |  |  |
| Over UNL | 20 (12.9%) | 14 (14.0%) | 6 (10.9%) |  |  |
| **CA125** |  |  |  | 0.765 |  |
| N | 135 (87.1%) | 86 (86.0%) | 49 (89.1%) |  |  |
| Over UNL | 20 (12.9%) | 14 (14.0%) | 6 (10.9%) |  |  |
| **CA199** |  |  |  | 1.000 |  |
| N | 138 (89.0%) | 89 (89.0%) | 49 (89.1%) |  |  |
| Over UNL | 17 (11.0%) | 11 (11.0%) | 6 (10.9%) |  |  |
| SD:standard deviation; LM: lymph node metastasis; N: normal; UNL: upper normal limit; CgA: chromogranin A; CK: Cytokeratin; NSE: neuron specific enolase; TAE: transarterial embolization; TACE: transcatheter hepatic arterial chemoembolization; LO: liver operation; PTR: primary tumor resection | | | | |  |
|  |  |  |  |  |  |

| **Supplementary table 2. Univariate and Multivariate Cox Regression Analysis for FAH Cohort with full characteristics** | | | | | | |  |
| --- | --- | --- | --- | --- | --- | --- | --- |
|  | **Univariate cox regression** | | | **Multivariate cox regression** | | |  |
| **Factors** | **HR** | **95%CI** | **P value** | **HR** | **95%CI** | **P value** |  |
| **Sex** |  |  |  |  |  |  |  |
| Female | Reference |  |  |  |  |  |  |
| male | 1.51 | 0.87-2.62 | 0.1441 |  |  |  |  |
| **Age** | 1.01 | 0.99-1.04 | 0.3899 |  |  |  |  |
| **Primary tumor site** |  |  |  |  |  |  |  |
| Stomach | Reference |  |  |  |  |  |  |
| Small Intestinal | 0.32 | 0.13-0.79 | 0.0134 |  |  |  |  |
| Colorectum | 0.54 | 0.29-0.99 | 0.0477 |  |  |  |  |
| **LM** |  |  |  |  |  |  |  |
| No |  |  |  |  |  |  |  |
| Yes | 0.96 | 0.43-2.12 | 0.9131 |  |  |  |  |
| **Tstage** |  |  |  |  |  |  |  |
| 1 | Reference |  |  |  |  |  |  |
| 2 | 0.83 | 0.35-2 | 0.6849 |  |  |  |  |
| 3 | 1.46 | 0.64-3.32 | 0.3629 |  |  |  |  |
| 4 | 2.13 | 0.95-4.78 | 0.0661 |  |  |  |  |
| **KI-67 index** |  |  |  |  |  |  |  |
| < 30 % | Reference |  |  | Reference |  |  |  |
| ≥ 30 % | 4.49 | 2.46-8.19 | <0.001 | 4.09 | 2.16-7.76 | <0.001 |  |
| **Grade** |  |  |  |  |  |  |  |
| NET-G1 | Reference |  |  |  |  |  |  |
| NET-G2 | 1.4 | 0.55-3.6 | 0.4825 |  |  |  |  |
| NET-G3 | 3.38 | 1.12-10.22 | 0.0313 |  |  |  |  |
| NEC | 6.08 | 3.6-18.3 | 0.0013 |  |  |  |  |
| **Primary tumor size** |  |  |  |  |  |  |  |
| <2 | Reference |  |  | Reference |  |  |  |
| >=2 | 3.17 | 1.35-7.42 | 0.0734 | 2.3 | 0.97-5.48 | 0.0601 |  |
| **Extrahepatic metastases** | |  |  |  |  |  |  |
| No | Reference |  |  | Reference |  |  |  |
| Yes | 2.5 | 1.46-4.28 | <0.001 | 2.21 | 1.18-4.15 | 0.0138 |  |
| **Liver tumor size** |  |  |  |  |  |  |  |
| <5cm | Reference |  |  |  |  |  |  |
| 5~10cm | 0.82 | 0.43-1.54 | 0.5306 |  |  |  |  |
| >10cm | 1.39 | 0.68-2.87 | 0.3664 |  |  |  |  |
| **Therapy of Liver** |  |  |  |  |  |  |  |
| **TAEorTACE** |  |  |  |  |  |  |  |
| No | Reference |  |  |  |  |  |  |
| Yes | 0.36 | 0.21-0.62 | <0.001 |  |  |  |  |
| **LO** |  |  |  |  |  |  |  |
| No | Reference |  |  |  |  |  |  |
| Yes | 0.53 | 0.22-1.24 | 0.1412 |  |  |  |  |
| **CD56** |  |  |  |  |  |  |  |
| Negative | Reference |  |  |  |  |  |  |
| Positive | 0.77 | 0.37-1.58 | 0.4723 |  |  |  |  |
| **CgA** |  |  |  |  |  |  |  |
| Negative | Reference |  |  |  |  |  |  |
| Positive | 0.93 | 0.54-1.6 | 0.7944 |  |  |  |  |
| **CK** |  |  |  |  |  |  |  |
| Negative | Reference |  |  |  |  |  |  |
| Positive | 1.57 | 0.7-3.51 | 0.2745 |  |  |  |  |
| **NSE** |  |  |  |  |  |  |  |
| N | Reference |  |  | Reference |  |  |  |
| Over UNL | 1.67 | 0.95-2.94 | 0.0753 | 1.85 | 1.02-3.33 | 0.0415 |  |
| **CA125** |  |  |  |  |  |  |  |
| N | Reference |  |  |  |  |  |  |
| Over UNL | 2.48 | 1.34-4.59 | 0.0038 | 2.08 | 1.07-4.05 | 0.0305 |  |
| **CA199** |  |  |  |  |  |  |  |
| N | Reference |  |  |  |  |  |  |
| Over UNL | 1.24 | 0.58-2.66 | 0.5846 |  |  |  |  |
| **CEA** |  |  |  |  |  |  |  |
| N | Reference |  |  |  |  |  |  |
| Over UNL | 0.99 | 0.47-2.12 | 0.984 |  |  |  |  |
| **PTR** |  |  |  |  |  |  |  |
| No | Reference |  |  | Reference |  |  |  |
| Yes | 0.49 | 0.27-0.88 | 0.0175 | 0.4 | 0.21-0.76 | 0.0049 |  |
| LM: lymph node metastasis; N: normal; UNL: upper normal limit; CgA: chromogranin A; CK: Cytokeratin; NSE: neuron specific enolase; TAE: transarterial embolization; TACE: transcatheter hepatic arterial chemoembolization; LO: liver operation; PTR: primary tumor resection; PTR: primary tumor resection; HR: hazard ration; CI: confidence interval. | | | | | | |  |
|  |  |  |  |  |  |  |  |

| **Supplementary Table 3. Comparison of Baseline Characteristics in pre-PSM Cohort Before and After IPTW Adjustment** | | | | | | | | |
| --- | --- | --- | --- | --- | --- | --- | --- | --- |
| **Characteristics** | **Before IPTW** | | | | **After IPTW** | | | |
|  | **Without PTR** | **PTR** | **P-value** | **SMD** | **Without PTR** | **PTR** | **P-value** | **SMD** |
|  | **(N=109)** | **(N=805)** |  |  | **(N=771.1)** | **(N= 906.9)** |  |  |
| **Age** |  |  | 0.176 | 0.070 |  |  | 0.186 | -0.103 |
| **<55** | 42 (38.5%) | 254 (31.6%) |  |  | 169.3 (22.0%) | 292.4 (32.2%) |  |  |
| **>=55** | 67 (61.5%) | 551 (68.4%) |  |  | 601.8 (78.0%) | 614.5 (67.8%) |  | 0.136 |
| **Sex** |  |  | 0.127 | 0.083 |  |  | 0.188 |  |
| **Female** | 62 (56.9%) | 391 (48.6%) |  |  | 488.2 (63.3%) | 450.5 (49.7%) |  |  |
| **Male** | 47 (43.1%) | 414 (51.4%) |  |  | 283.0 (36.7%) | 456.5 (50.3%) |  |  |
| **PTS** |  |  | <0.001 |  |  |  | 0.625 |  |
| **Stamoch** | 25 (22.9%) | 20 (2.5%) |  | -0.205 | 42.3 ( 5.5%) | 42.3 ( 4.7%) |  | -0.008 |
| **Small intestine** | 22 (20.2%) | 674 (83.7%) |  | 0.635 | 558.3 (72.4%) | 695.8 (76.7%) |  | 0.043 |
| **Colorectum** | 62 (56.9%) | 111 (13.8%) |  | -0.431 | 170.5 (22.1%) | 168.8 (18.6%) |  | -0.035 |
| **Grade** |  |  | <0.001 |  |  |  | 0.889 |  |
| **Well differentiation G1** | 23 (21.1%) | 488 (60.6%) |  | 0.395 | 454.6 (58.9) | 516.3 (56.9) |  | -0.020 |
| **Moderate differentiation G2** | 11 (10.1%) | 166 (20.6%) |  | 0.105 | 132.4 (17.2) | 179.6 (19.8) |  | 0.026 |
| **Poor differentiation G3** | 48 (44.0%) | 92 (11.4%) |  | -0.326 | 123.2 (16.0) | 128.7 (14.2) |  | -0.018 |
| **NEC** | 27 (24.8%) | 59 (7.3%) |  | -0.174 | 61.1 ( 7.9) | 82.5 ( 9.1) |  | 0.012 |
| **T** |  |  | <0.001 |  |  |  | 0.215 |  |
| **T1** | 10 (9.2%) | 18 (2.2%) |  | -0.069 | 56.0 ( 7.3) | 26.1 ( 2.9) |  | -0.044 |
| **T2** | 45 (41.3%) | 91 (11.3%) |  | -0.300 | 202.5 (26.3) | 134.2 (14.8) |  | -0.115 |
| **T3** | 25 (22.9%) | 392 (48.7%) |  | 0.258 | 256.0 (33.2) | 415.7 (45.8) |  | 0.126 |
| **T4** | 29 (26.6%) | 304 (37.8%) |  | 0.112 | 256.7 (33.3) | 331.0 (36.5) |  | 0.032 |
| **Primary tumor size** | |  | <0.001 | -0.217 |  |  | 0.542 | -0.056 |
| **<2** | 8 (7.3%) | 234 (29.1%) |  |  | 165.1 (21.4%) | 244.8 (27.0%) |  |  |
| **>=2** | 101 (92.7%) | 571 (70.9%) |  |  | 606.1 (78.6%) | 662.1 (73.0%) |  |  |
| **Extrahepatic metastases** | |  | <0.001 | -0.236 |  |  | 0.973 | -0.001 |
| **No** | 78 (71.6%) | 766 (95.2%) |  |  | 717.3 (93.0%) | 844.2 (93.1%) |  |  |
| **Yes** | 31 (28.4%) | 39 (4.8%) |  |  | 53.9 (7.0%) | 62.7 (6.9%) |  |  |

| **Supplementary Table 4. Univariate and Multivariate Cox Regression Analysis Before and After IPTW Adjustment in pre-PSM SEER Cohort** | | | | | | | | |
| --- | --- | --- | --- | --- | --- | --- | --- | --- |
|  | **Before IPTW weighted** | | | | **After IPTW weighted** | | | |
|  | **Univariate cox regression** | | **Multivariate cox regression** | | **Univariate cox regression** | | **Multivariate cox regression** | |
|  | **HR （95%CI）** | **P value** | **HR （95%CI）** | **P value** | **HR （95%CI）** | **P value** | **HR （95%CI）** | **P value** |
| **Age** |  |  |  |  |  |  |  |  |
| **<55** | Reference |  | Reference |  | Reference |  | Reference |  |
| **>=55** | 1.75 (1.41-2.18) | <0.001 | 1.74(1.39-2.17) | <0.001 | 1.37 (0.93-2.01) | 0.111 | 1.33(0.97-1.84) | 0.078 |
| **Sex** |  |  |  |  |  |  |  |  |
| **Female** | Reference |  | Reference |  | Reference |  |  |  |
| **Male** | 1.16 (0.96-1.4) | 0.120 | 1.26(1.05-1.53) | 0.015 | 1.04 (0.65-1.68) | 0.857 |  |  |
| **PTS** |  |  |  |  |  |  |  |  |
| **Stamoch** | Reference |  | Reference |  | Reference |  | Reference |  |
| **Small intestine** | 0.22 (0.15-0.3) | <0.001 | 0.59(0.39-0.88) | 0.011 | 0.28 (0.17-0.47) | <0.001 | 0.68(0.39-1.18) | 0.170 |
| **Colorectum** | 1.12 (0.64-1.5) | 0.516 | 1.06(0.73-1.53) | 0.769 | 1.22 (0.8-1.87) | 0.350 | 1.23(0.72-2.11) | 0.439 |
| **Grade** |  |  |  |  |  |  |  |  |
| **Well differentiation G1** | Reference |  | Reference |  | Reference |  | Reference |  |
| **Moderate differentiation G2** | 1.69 (1.29-2.21) | <0.001 | 1.66(1.27-2.17) | <0.001 | 2.37 (1.27-4.43) | 0.007 | 2.42(1.31-4.44) | 0.005 |
| **Poor differentiation G3** | 11.63 (9.1-14.87) | <0.001 | 6.98(5.18-9.4) | <0.001 | 10.08 (6.08-16.69) | <0.001 | 7.48(4.24-13.22) | <0.001 |
| **NEC** | 9.57 (7.9-12.71) | <0.001 | 6.13(4.43-8.49) | <0.001 | 9.18 (5.1-16.52) | <0.001 | 6.52(3.51-12.11) | <0.001 |
| **T** |  |  |  |  |  |  |  |  |
| **T1** | Reference |  | Reference |  | Reference |  | Reference |  |
| **T2** | 0.74 (0.43-1.25) | 0.261 | 0.47(0.27-0.82) | 0.008 | 1.49 (0.49-4.54) | 0.482 | 0.9(0.25-3.23) | 0.870 |
| **T3** | 0.60 (0.37-0.99) | 0.044 | 0.59(0.34-1.01) | 0.056 | 1.62 (0.59-4.45) | 0.346 | 1.16(0.39-3.45) | 0.783 |
| **T4** | 0.78 (0.47-1.28) | 0.324 | 0.69(0.4-1.21) | 0.197 | 2.05 (0.68-6.16) | 0.201 | 1.24(0.38-4.02) | 0.721 |
| **PTR** |  |  |  |  |  |  |  |  |
| **No** | Reference |  | Reference |  | Reference |  | Reference |  |
| **Yes** | 0.24 (0.19-0.30) | <0.001 | 0.62(0.46-0.83) | 0.002 | 0.41 (0.23-0.71) | 0.037 | 0.51(0.34-0.77) | 0.002 |
| **Primary tumor size** |  |  |  |  |  |  |  |  |
| **<2** | Reference |  | Reference |  | Reference |  | Reference |  |
| **>=2** | 2.01 (1.58-2.56) | <0.001 | 1.26(0.96-1.66) | 0.095 | 2.79 (1.46-5.33) | 0.005 | 1.35(0.66-2.78) | 0.413 |
| **Extrahepatic metastases** |  |  |  |  |  |  |  |  |
| **No** | Reference |  | Reference |  | Reference |  | Reference |  |
| **Yes** | 3.62 (2.75-4.77) | <0.001 | 1.54(1.14-2.09) | 0.005 | 2.44 (1.49-3.98) | <0.001 | 1.44(1.02-2.05) | 0.040 |
